# Structure‐based design of ferritin nanoparticle immunogens displaying antigenic loops of *Neisseria gonorrhoeae*

**DOI:** 10.1002/2211-5463.12267

**Published:** 2017-07-24

**Authors:** Liqin Wang, Daniel Xing, Adriana Le Van, Ann E. Jerse, Shuishu Wang

**Affiliations:** ^1^ Department of Biochemistry and Molecular Biology Uniformed Services University of the Health Sciences Bethesda MD USA; ^2^ Department of Microbiology and Immunology Uniformed Services University of the Health Sciences Bethesda MD USA

**Keywords:** crystallography, gonorrhea, nanoparticle, rational vaccine design, structural vaccinology

## Abstract

Effective vaccines are urgently needed to combat gonorrhea, a common sexually transmitted bacterial infection, for which treatment options are diminishing due to rapid emergence of antibiotic resistance. We have used a rational approach to the development of gonorrhea vaccines, and genetically engineered nanoparticles to present antigenic peptides of *Neisseria gonorrhoeae*, the causative agent of gonorrhea. We hypothesized that the ferritin nanocage could be used as a platform to display an ordered array of *N. gonorrhoeae* antigenic peptides on its surface. MtrE, the outer membrane channel of the highly conserved gonococcal MtrCDE active efflux pump, is an attractive vaccine target due to its importance in protecting *N. gonorrhoeae* from host innate effectors and antibiotic resistance. Using computational approaches, we designed constructs that expressed chimeric proteins of the *Helicobacter pylori* ferritin and antigenic peptides that correspond to the two surface‐exposed loops of *N. gonorrhoeae* MtrE. The peptides were inserted at the N terminus or in a surface‐exposed ferritin loop between helices αA and αB. Crystal structures of the chimeric proteins revealed that the proteins assembled correctly into a 24‐mer nanocage structure. Although the inserted *N. gonorrhoeae* peptides were disordered, it was clear that they were displayed on the nanocage surface, but with multiple conformations. Our results confirmed that the ferritin nanoparticle is a robust platform to present antigenic peptides and therefore an ideal system for rational design of immunogens.

**Abbreviations**
EM: electron microscopy
HIV: human immunodeficiency virus
Hpf: *Helicobacter pylori* ferritin
NCS: noncrystallographic symmetry
*Ng*: *Neisseria gonorrhoeae*
rmsd: root mean square deviation

Effective vaccines are still needed for many human pathogens that significantly impact global health. Gonorrhea has a significant impact on reproductive and neonatal health worldwide, and it is a known cofactor in the spread of human immunodeficiency virus (HIV). Over 106 million *Neisseria gonorrhoeae* infections are estimated worldwide annually 1, and gonorrhea is the second most commonly reported notifiable infectious disease in the United States. The rapid emergence of antibiotic resistance in *N. gonorrhoeae* has prompted a resurgence in research in gonorrhea vaccine development 2, which currently is at the level of antigen discovery and the identification of protective immune responses. While antigenic variability of surface molecules has frustrated gonorrhea vaccine development, several conserved or semiconserved antigens, such as PorB and MtrE, have since been identified as promising vaccine targets 3, 4, 5.

MtrE is a highly conserved outer membrane channel of three efflux pumps 6, MtrCDE, MacA‐MacB‐MtrE, and FarA‐FarB‐MtrE. These efflux pumps play important roles in *N. gonorrhoeae* pathogenesis and antibiotic resistance 4, 7, 8, 9, 10, 11. The MtrE protein is a trimer; each subunit has two short surface‐exposed loops that extend from the strands of the outer membrane β‐barrel 12 and are highly conserved in all *N. gonorrhoeae* strains. These surface‐exposed loops are antigenic. Antibodies against a linear peptide of MtrE loop 1 recognize the MtrE protein in western blots 4, and antibodies against a recombinant MtrE are bactericidal and bind to *N. gonorrhoeae* cell surface 13. Use of the whole MtrE molecule as a vaccine antigen is not an optimal choice because the bulk of the molecule is non‐surface‐exposed that can thereby divert much of the immune response from the surface‐exposed loops. In addition, purification of the whole MtrE protein and maintaining its native conformation are challenging because it is a membrane protein and requires detergent to stay in solution. The use of soluble peptide antigens to mimic the surface‐exposed loops is challenged by the relatively short sequence of each loop, which limits the immunogenicity of the peptides, and the possibility that conformational epitopes may not be reproduced by linear peptides. To overcome these challenges, there is a need for a new vaccine development platform that can strategically present various antigenic peptides and allow rapid and systematic screening of various candidate vaccines for those that present antigen that induces strong immune responses. Recent developments in structural vaccinology provide a promising approach for such vaccine platforms 14. One of these novel technologies is to display structurally defined antigenic epitopes in high copy numbers on the surface of self‐assembling nanoparticles, such as virus‐like particles or protein nanocages. Ferritin, a protein that self‐assembles into a cage of 24 identical subunits, is a suitable antigen‐presenting platform 15. The protein is a large enough multi‐subunit nanoparticle that is relatively stable and amenable to crystal structure determination. Moreover, it was demonstrated that large protein domains could be fused to the N terminus of ferritin without disrupting the assembly of the nanocage structure 16, 17.

In this study, we investigated the feasibility of using the *Helicobacter pylori* ferritin (Hpf) nanoparticle to present *N. gonorrhoeae* antigenic surface‐exposed loops of the MtrE protein with the long‐term goal of producing effective, rationally designed gonorrhea vaccines. We inserted the two surface loops of MtrE into the Hpf sequence and showed that the MtrE loop‐Hpf chimeras assembled into the 24‐mer cage structure. Although the MtrE loops are disordered, the crystal structures indicated that the loops are displayed on the nanoparticle surface in a way that should be accessible to antibody binding. These structures can guide further design of the nanoparticles to those that display the antigenic loops in a conformation that maximizes the immunogenicity.

## Results

### Computational design of constructs

**Figure 1:**
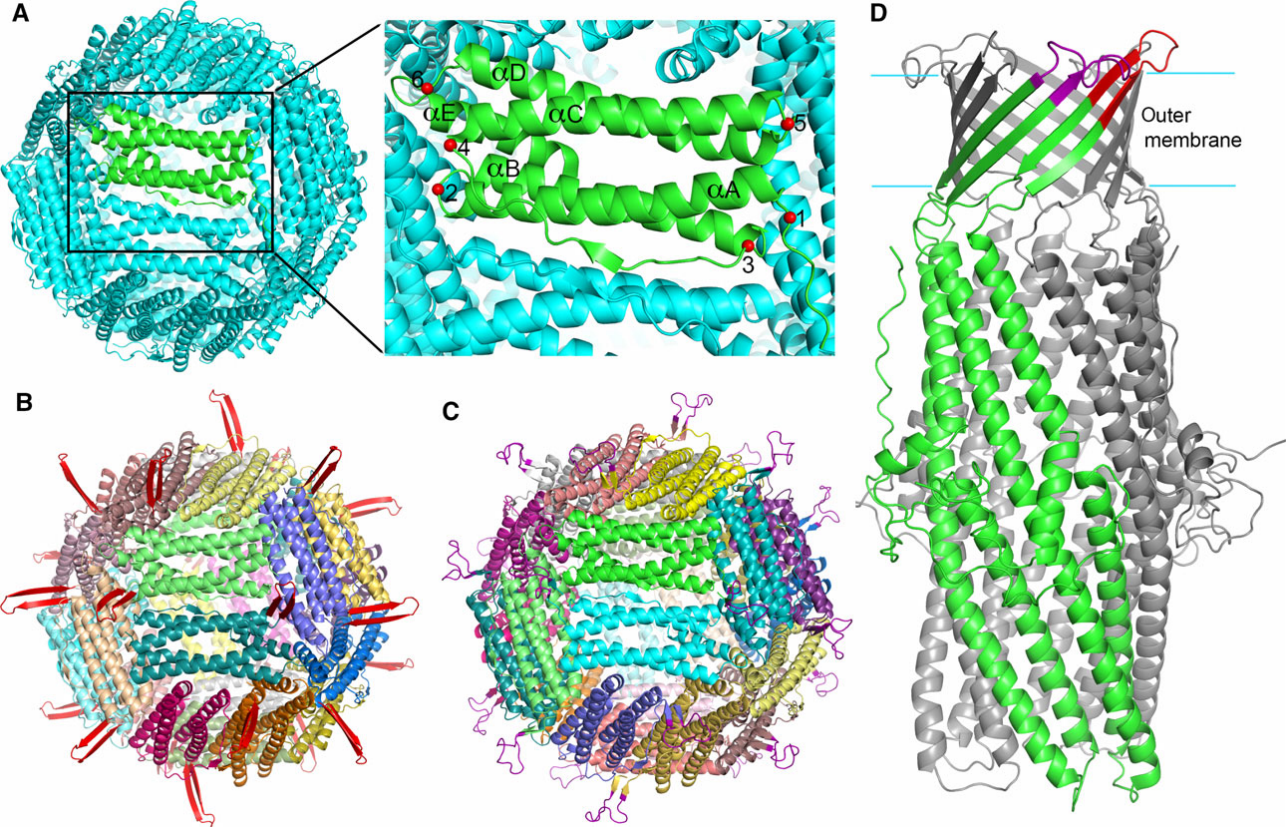
Computational design of the Hpf‐loop nanoparticles. (A) Hpf structure and potential insertion sites for antigenic *N. gonorrhoeae* peptides on the structure of ferritin. The Hpf structure (Pdb code 3BVF) is shown in cartoon representation. One subunit is colored in green, while the rest in cyan. The potential insertion sites for *N. gonorrhoeae* peptides are marked on the green subunit with red spheres and numbered from 1 to 6. Helices αA to αE are labeled. (B) Computational model of an Hpf‐E1 nanoparticle. The MtrE loop 1 (colored red) is inserted at site 2, replacing His34. A two‐stranded β‐sheet projects the loop outward from the surface. (C) Computational model of Hpf‐E2. The MtrE loop 2 (colored purple) is inserted at site 1, the N terminus of Hpf. (D) Cartoon representation of the MtrE structure (Pdb code 4mt0) showing the peptides loop 1 (colored red) and loop 2 (colored purple) that were selected to build Hpf‐E1 and Hpf‐E2, respectively. One subunit of MtrE is colored in green with the surface loops marked, and the other two subunits in gray.

**Figure 2:**
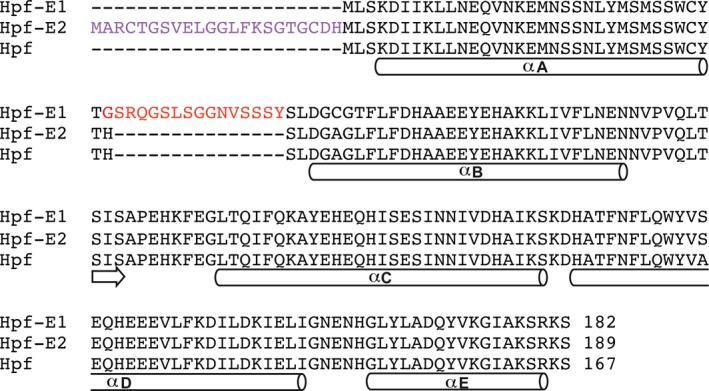
Sequence alignment showing the inserted peptide sequences of Hpf‐E1 and Hpf‐E2 relative to the Hpf sequence. Sequence segments that form α‐helices are marked with a cylinder below the sequences. A short sequence that has inter‐subunit β‐strand hydrogen bonds with a neighboring subunit is also marked with an arrow. The MtrE loop 1 peptide is inserted in Hpf‐E1 after Thr33, with the inserted peptide sequence highlighted in red. In Hpf‐E2, the MtrE loop 2 peptide is inserted at the N terminus (inserted sequence highlighted in purple), flanked by two Cys residues, which if form a disulfide will restrict the peptide to fold into a β‐hairpin loop with correct register of the cross‐strand hydrogen bonds.

We postulated that antigenic loop peptides of *N. gonorrhoeae* can be displayed on the surface of Hpf by rational design of chimeric proteins. To test this idea, we constructed chimeric MtrE loop‐Hpf proteins computationally by inserting the structural fragments of the two extracellular MtrE loops 12 into the Hpf structure. The Hpf structure is a 24‐mer cage, each subunit of which consists of five α‐helices (αA to αE) 18. Using computer 3D graphics, we copied the structural fragments containing extracellular loops from the crystal structure of MtrE 12 and inserted them at the desired locations of the Hpf structure (Fig. 1). Two structural models were constructed: one with MtrE loop 1 inserted at the loop between Hpf helices αA and αB (the construct is designated as Hpf‐E1) and the other with MtrE loop 2 at the N terminus of Hpf (designated as Hpf‐E2) (Fig. 2). The manually built models were energy‐minimized with 3Drefine 19 to correct any deviations of the main‐chain and side‐chain geometry of the inserted residues. These structural models were the bases for generating the DNA constructs for the expression and purification of the MtrE loop‐Hpf chimeric proteins as described below.

### Protein purification and analysis

**Figure 3:**
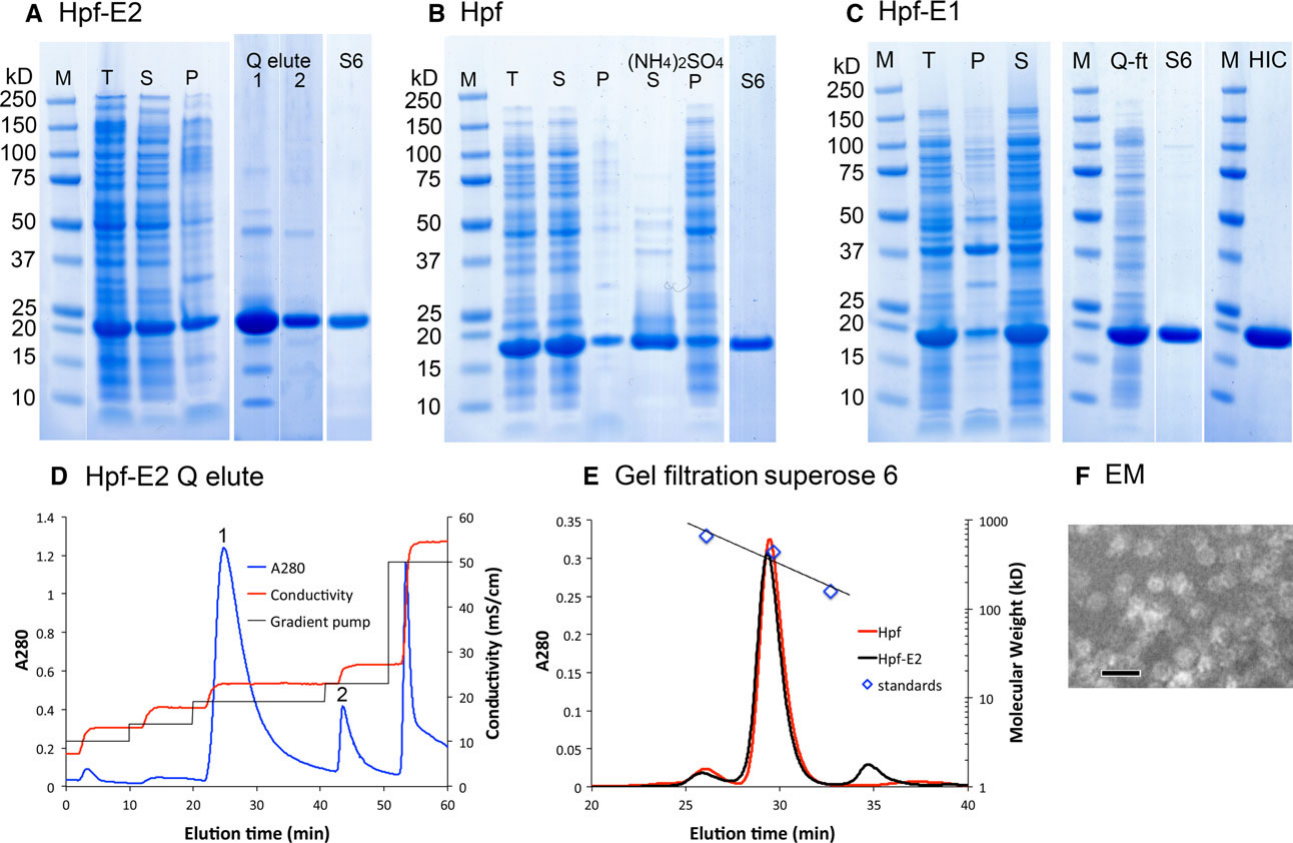
Protein purification and characterization. Panels (A), (B), and (C) are SDS/PAGE of Hpf‐E2, Hpf, and Hpf‐E1, respectively, stained by SimplyBlue (Invitrogen, Carlsbad, CA, USA). Lanes marked M are markers; T, total cell lysate; S, supernatant; P, pellets. Lanes marked Q elute 1 and 2 are from peaks marked 1 and 2 in (D). Lanes marked S6 are after the Superose 6 gel filtration as shown in (E). Lanes in (B) marked (NH_4_)_2_SO_4_ S and P are the supernatant and pellet fractions of ammonium sulfate precipitation (65% saturation) of the cell lysate supernatant. In panel (C), the lane marked Q‐ft is the flow‐through from the loading of a HiTrap Q column in 20 mm Tris pH 8.0 and 50 mm NaCl, and lane HIC is the protein after hydrophobic interaction chromatography using a butyl HP column. A minor contaminant ~ 100 kDa on S6 was removed by HIC. (D) Chromatogram of a HiTrap Q column elution of Hpf‐E2. (E) Gel filtration chromatograms. The standards are thyroglobulin (669 kDa), ferritin (440 kDa), and aldolase (158 kDa), from the Gel Filtration HMW Calibration Kit (GE). The calculated molecular weight of the Hpf‐E2 nanoparticle is 514 kDa, and that for the wild‐type Hpf is 464 kDa. (F) An electron micrograph of purified Hpf‐E2. The scale bar is 20 nm. The EM images of Hpf and Hpf‐E1 are identical.

Based on the designed loop‐nanoparticle structures, DNA sequences encoding the polypeptides were generated and codon‐optimized for expression in *Escherichia coli*. No affinity tag was present in the expressed proteins, and the proteins were purified by ammonium sulfate precipitation, followed by ion‐exchange and hydrophobic interaction chromatography (Fig. 3). The proteins were then further purified and buffer‐exchanged by gel filtration prior to crystallization. Gel filtration chromatography indicated that the proteins were of the expected size for a 24‐mer ferritin nanoparticle (Fig. 3E). The spherical feature of the nanoparticles of the purified chimeras was confirmed by electron microscopy (EM; Fig. 3F). The Hpf‐E1 protein was expressed in *E. coli* as a soluble protein. However, a large fraction of the Hpf‐E2 protein expressed in *E. coli* was in inclusion bodies. The inclusion bodies were solubilized in 8 m urea, and the protein refolded by removing urea through dialysis. The refolded and the soluble Hpf‐E2 proteins behaved identically in chromatography and gave identical electron micrographs. The refolded Hpf‐E2 protein was used to obtain the crystal. In contrast to Hpf‐E2, which is a fusion of MtrE loop 2 to the N terminus, Hpf‐E1 has the MtrE loop 1 inserted in the middle of the Hpf sequence. Successful production of both proteins demonstrated that it is feasible to insert *N. gonorrhoeae* antigenic peptides at the N terminus or in selected locations in the middle of Hpf sequence without perturbing the nanocage structure.

### Overall crystal structure of Hpf‐E1

To confirm that the chimeric proteins indeed formed the correct nanocage structures and that the *N. gonorrhoeae* peptides were displayed on the surface, we determined the crystal structures of Hpf‐E1 and Hpf‐E2. The Hpf‐E1 crystal structure was determined to 2.0 Å. The data and structural refinement statistics are listed in Table 1. The crystal was in the space group P4. There were 12 subunits in the asymmetric unit (designated as A to L). The 12 subunits were grouped into two hexamers (A to F and G to L) that are related by translational noncrystallographic symmetry (NCS). Each hexamer was operated on by the crystallographic fourfold symmetry to generate a 24‐mer spherical cage. Each cage has a total solvent‐accessible surface area of ~ 148 000 Å^2^ and a total area buried in the inter‐subunit interfaces of ~ 74 000 Å^2^. The two cages had identical packing of the subunits. By aligning one subunit, the Cα traces of all subunits of the two cages superposed perfectly.

**Table 1:** X‐ray diffraction data and refinement statistics

Data		
Crystal	Hpf‐E1	Hpf‐E2
Resolution (Å)^a^	20–2.0 (2.03–2.00)	20–2.8 (2.85–2.80)
Space group	P4	H3
Unit cell (a, b, c) (Å)	128.4, 128.4, 165.0	124.5, 124.5, 314.9
*R*_sym_ (%)^a^	13.8 (89.5)	16.6 (79.6)
*R*_pim_ (%)^a^	6.4 (42.3)	8.8 (42.1)
Completeness (%)^a^	99.8 (100.0)	93.4 (99.9)
Mean I/σ (I)^a^	14.6 (2.0)	10.1 (1.7)
Redundancy^a^	5.6 (5.4)	4.6 (4.5)
Refinement		
Resolution (Å)^a^	20–2.0 (2.05–2.00)	20–2.8 (2.88–2.81)
*R*_work_ a b	0.181 (0.267)	0.177 (0.214)^c^
*R*_free_ a b	0.204 (0.302)	0.225 (0.291)^c^
rmsd (bond) (Å)	0.016	0.018
rmsd (angle) (°)	1.755	1.653
Ramachandran plot^d^	99% favored, 1% allowed	97% favored, 3% allowed
Number of peptide chains per asymmetric unit	12	8
Number of protein atoms (ave. B factor, Å^2^)	16 447 (27.0)	10 884 (62.6)
Number of waters (ave. B factor, Å^2^)	1398 (35.9)	11 (38.6)
Other molecules (ave. B factor, Å^2^)	30 Na^+^ (34.3), 6 Fe^3+^ (39.7), 12 glycerol (44.6), 8 Cl^−^ (30.0)	
PDB ID	5U1A	5U1B

a The values for the data in the highest resolution shell are shown in parentheses.

b *R*_work_ was calculated on the reflections used in the refinement; *R*_free_ was calculated on a set of approximately 5% randomly chosen reflections that were never used for the refinement.

c The Hpf‐E2 was twinned and two twin domains with fractions of approximately 0.544 and 0.456 were used in the refinement by REFMAC and for the calculation of the R factors.

d Ramachandran plot statistics were calculated with wwPDB validation server (https://validate.wwpdb.org/validservice/).

The crystals were grown in the presence of 1.6 m NaCl and 20% glycerol, and at least 30 Na^+^ and 8 Cl^−^ ions, and 12 glycerol molecules were found in the asymmetric unit (Table 1). Na^+^ and Cl^−^ ions were assigned based on their coordination and favorable charged environments. Na^+^ tends to have octahedral coordination with hydrogen bond acceptor groups at ~ 2.4 Å distances. Cl^−^ interacts with hydrogen bond donors at distances of ~ 3.0–3.4 Å. In addition, six Fe^3+^ ions (although modeled as Fe^3+^ in the structure, the oxidation state of the ion is unclear, and from here on, it is referred to as Fe) were modeled in the structure at the fourfold symmetry vertex, each coordinated by four histidine side chains (His164 in Hpf‐E1) in a plane and two water molecules at the axial positions. The same sites are occupied by Fe ions in the Hpf structure 18. Four of the Fe ions were at the fourfold crystallographic symmetry axes. The electron density fit well with Fe with B factors of 38–43 Å^2^; modeling them as water or Na^+^ would result in a large peak of residual positive electron density. Because no Fe was added in the purification and crystallization process, the Fe ions were likely to be incorporated while the protein was synthesized in *E. coli* cells.

No Fe was modeled at the ferroxidase center, where two Fe ions are expected to bind next to each other and be coordinated by the side chains of four Glu, one Gln, and one His 18. Instead, one Na^+^ ion was modeled at the stronger Fe site coordinated by Glu65, Glu109, Gln142, and Glu145 (Glu50, Glu94, Gln127, and Glu130 in the native Hpf sequence). The B factor for the Na^+^ was ~ 23 Å^2^, similar to those of the coordinating atoms. The side chain of Glu65 had slightly elevated B factors (~ 35 Å^2^), which was due to multiple slightly different possible conformations as indicated by the contour of the electron density. The second Fe‐binding site was modeled as a water because the hydrogen bonds are consistent with a water. However, this could possibly be a Na^+^ because of the highly electronegative environment. The loss of Fe at the ferroxidase center could be due to the high concentration of NaCl in the crystallization condition.

### Overall crystal structure of Hpf‐E2

The Hpf‐E2 structure was determined at a lower resolution of 2.8 Å. The crystal was in the space group H3 with eight polypeptides (designated as A to H) in the asymmetric unit. The threefold crystallographic symmetry operated on the asymmetric unit content to generate a 24‐mer cage. The packing of the Hpf‐E2 subunits in the nanocage structure was identical to that of the Hpf‐E1, with minor shifts in relative positions (see alignments of subunits below for details). Because of the relatively low resolution, only 11 water molecules and no other ions/small molecules were modeled (Table 1). The Hpf‐E2 protein was purified from refolding of inclusion bodies. Therefore, there were no endogenously bound metal ions, such as Fe, identified in the crystal structure. The His side chains that coordinated the Fe in Hpf‐E1 had a slight shift away from each other in Hpf‐E2.

### Location of the inserted MtrE loops

**Figure 4:**
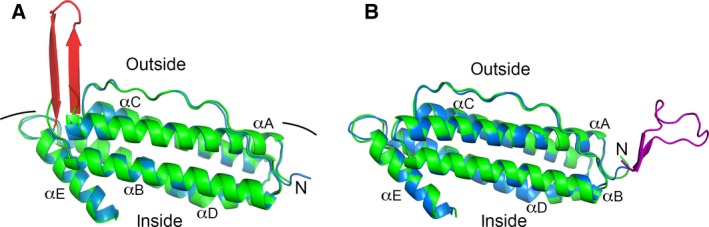
Superposition of Hpf‐E1 (A) and Hpf‐E2 (B) crystal structures with their corresponding computational models. For both crystal structures, subunit A was used in the structural alignment using the COOT SSM protocol. The crystal structures are colored green, and the computational models are in blue except the inserted peptides, which are in red and purple for Hpf‐E1 and Hpf‐E2, respectively. The molecules are oriented so that the outside surface of the nanocage is on top and the inside is at the bottom. The amino (N) terminus and helices αA to αE are labeled. Both crystal structures superposed almost perfectly with their computational models. Although the inserted *N. gonorrhoeae* peptides were disordered, it is clear that the peptides were displayed on the surface of the nanocage.

**Figure 5:**
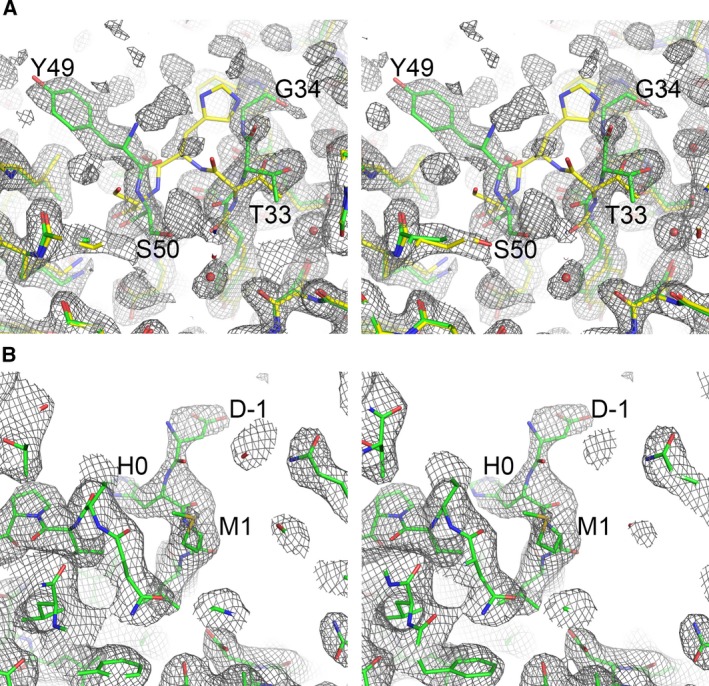
Stereo diagrams of electron density maps showing density for part of the inserted peptides in Hpf‐E1 (A) and Hpf‐E2 (B). Sections of the electron density maps around the inserted peptides were plotted at a contour level of 1 σ. The maps were calculated using REFMAC, with the inserted peptide residues omitted, at 2.0 Å in (A) and at 2.8 Å in (B). The refined structural models were colored in green. In (A), the Hpf structure, shown in yellow, was superimposed on the refined model. The inserted peptide residues and the immediate border residues of Hpf were labeled.

The packing of the 24‐mer cage structure of both Hpf‐E1 and Hpf‐E2 was identical to that of the wild‐type Hpf. However, the inserted *N. gonorrhoeae* peptides in both structures were disordered. To determine the location and potential conformations of the inserted *N. gonorrhoeae* peptides, we superposed the Hpf‐E1 subunit structure with the computationally designed model (Fig. 4A). The Hpf‐E1 structure was essentially identical to the computational model. Most of the subunits of Hpf‐E1 had some weak electron density for part of the inserted peptide that suggested that the MtrE loop 1 peptide adopted multiple conformations (Fig. 5A). It is likely that the *N. gonorrhoeae* peptide could have conformations that did not fold into its native β‐hairpin structure. However, the conformations of the ordered or partially ordered residues flanking the inserted peptide indicated that the MtrE loop 1 peptide was displayed on the surface of the nanocage.

Similar to the Hpf‐E1 structure, in the Hpf‐E2 structure the residues of the inserted MtrE loop 2 peptide were mostly disordered. Only subunits A and H had some weak density for two residues before the first Met of the Hpf sequence (Fig. 5B). The Hpf‐E2 structure was similar to the Hpf structure with an rmsd of 0.43 Å for Cα atoms. Superposition of the crystal structure with the computationally designed model indicated that the structure was as designed (Fig. 4B). It is clear from the crystal structure that the peptide must be displayed on the outside surface of the Hpf nanocage, but with multiple conformations.

The disordering of the inserted peptides is likely due to the intrinsic flexibility of the peptide. Although the peptides form β‐hairpins in the context of the whole MtrE protein, which folds into a stable β‐barrel, they are too flexible to have a single stable structure when taken out of the structural context and the outer membrane environment. Nevertheless, the insertion of the MtrE loop peptides did not perturb the Hpf structure, confirming the feasibility of using Hpf as a platform for developing candidate immunogens that present an ordered array of the *N. gonorrhoeae* antigenic peptides on the surface.

### Comparison of the structures and temperature factors of the subunits

**Figure 6:**
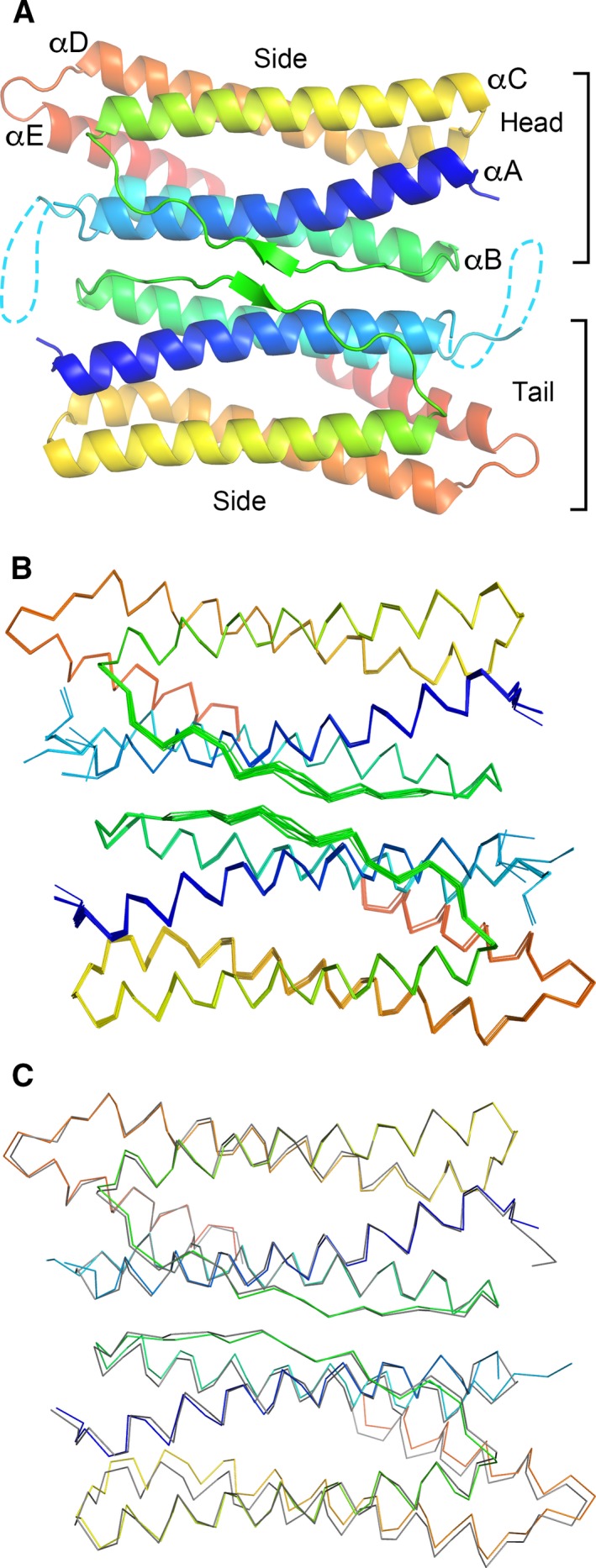
Hpf‐E1 dimer with the twofold symmetry interface. (A) Ribbon diagram of the twofold symmetric dimer. The diagram is oriented looking down from the exterior of the nanocage along the twofold symmetry axis. Each subunit is colored rainbow from blue to red for N to C termini, respectively. The disordered *N. gonorrhoeae* peptide is depicted as a dashed line. There are two unique edges of the dimer that are involved in the nanocage packing. One is designated as the side, and the other includes the ends of the helical bundle, designated as head for the end with the N terminus and as tail for the other end. (B) Structural alignments of all subunits in the asymmetric unit. Only the top subunit of the dimer is aligned pairwise against subunit A. The bottom subunit has a slight shift against each other, with a larger shift for helices αD and αE that are away from the dimer interface. Residues flanking the inserted *N. gonorrhoeae* peptide adopt various conformations among the subunits. (C) Structural alignment between subunit A of Hpf‐E1 and that of Hpf‐E2. The Hpf‐E1 structure is shown in rainbow color, and the Hpf‐E2 is shown in gray. Only the top subunits of the dimer are aligned. The shift between the two structures in the bottom subunit of the dimer is slightly larger than that between the subunits of Hpf‐E1.

To identify ways to improve the stability and robustness of the Hpf antigen‐presenting platform, we compared the subunits for their structural differences and temperature factor variations. The highly flexible regions could be chosen as the locations for inserting *N. gonorrhoeae* peptides or targeted for mutagenesis to improve the structural stability. The 12 subunits in the asymmetric unit of the Hpf‐E1 crystal had a pairwise rmsd for Cα atoms ranging from ~ 0.25 to 0.35 Å. Most differences were in the long BC loop, which connects helices αB and αC (Fig. 6). Other regions with significant deviations in the main‐chain atom positions among some subunits were at the first five residues at the N terminus, residues 11–16 in helix αA, residues 121–130 in and around loop CD, and residues 160–168 in and around loop DE. These regions had higher B factors, indicating their flexibility. It is noteworthy that the DE loop, which harbors the His side chain (His164 in Hpf‐E1) that coordinates the Fe ion at the fourfold symmetry channel, is highly flexible.

### Molecular packing interfaces of the nanocage structure

**Figure 7:**
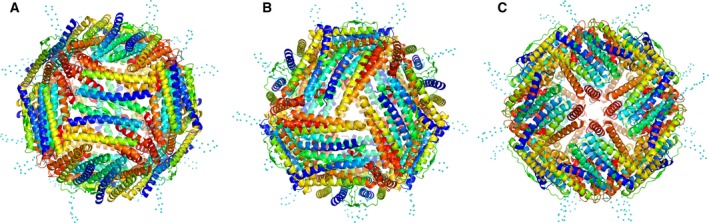
Cartoon representations of the spherical cage structure of Hpf‐E1. The diagrams are oriented to show the face at the twofold (A), threefold (B), and fourfold (C) symmetry axes. Each subunit of Hpf‐E1 is colored rainbow, with blue for the N terminus and red for the C terminus. The cyan dotted lines mark the approximate locations of the inserted MtrE loop 1 peptide. Although the peptides are disordered in the structure, their locations indicate that they are projected from the surface of the nanocage.

The packing of the 24‐mer nanocage involved three different subunit interfaces (Table 2). On average, each subunit had a total surface area of ~ 9227 Å^2^ and a total buried surface area in the interfaces of ~ 3341 Å^2^. The largest interface between two subunits was at where the two subunits were related by the twofold symmetry (Table 2). This interface buried an average of 1203 Å^2^, more than a third of the total interface area, and involved helices αA and αB and the BC loop (Fig. 6A). The interface involved mostly polar interactions, including 12–17 hydrogen bonds and charge–charge interactions among Glu67 and Lys70 side chains of both subunits near the twofold axis. There were also aromatic interactions between two Tyr66, hydrophobic interactions between two Ile88 side chains at the center, and hydrophobic interactions among side chains of Phe59 and Met25 of one subunit and Ile73, Val74, and Lys70 of the other. The overall assembly of the nanocage can be described as the packing of this twofold symmetry dimer (Fig. 7). Because of the twofold symmetry, the dimer has only two unique edges that are involved in the nanocage packing: One is the side along αC and αD and the other includes the ends of the helical bundle, designated as head for the end with the N terminus and as tail for the other end (Fig. 6A).

**Table 2:** Buried area in the inter‐subunit interfaces of Hpf‐E1

Subunits	Interface^a^	Area^b^ (Å^2^)
K–H	Twofold dimer	1248.1
E–B	Twofold dimer	1213.3
D–C	Twofold dimer	1195.1
J–I	Twofold dimer	1193.1
L–G	Twofold dimer	1189.1
F–A	Twofold dimer	1179.4
G–L	Head to side	546.7
C–F	Head to side	542.9
A–F	Head to side	528.8
I–G	Head to side	504.8
C–A	Head to side	498.0
I–L	Head to side	484.6
E–B	Head to side	469.4
E–D	Head to side	468.5
K–H	Head to side	459.5
D–B	Head to side	457.9
K–J	Head to side	456.7
J–H	Head to side	453.1
D–B	Tail to side	462.7
F–F	Tail to side	454.4
D–A	Tail to side	440.7
K–K	Tail to side	435.0
E–E	Tail to side	434.9
L–L	Tail to side	433.1
I–H	Tail to side	432.3
C–A	Tail to side	432.1
J–G	Tail to side	430.9
I–G	Tail to side	422.3
C–B	Tail to side	422.3
J–H	Tail to side	420.5

a Side is defined as the edge along helical axis on the side of helices αC, αD, and αE, away from the twofold symmetric dimer interface; head is the end of a subunit with the N terminus; tail is the other end of the subunit with loop DE and the C terminus of helix αA.

b Differences in buried areas of the same type interface are due to disordered residues and/or side chains.

The dimer–dimer interactions involved only one type of interface: the side of one dimer packed against the edge of head‐and‐tail of another. There were two types of inter‐subunit interfaces involved in this dimer–dimer interface. The head of one subunit (the end with the N terminus) interacted with the middle regions of αC and αD of another, which covered an average of ~ 500 Å^2^ (Table 2). Three such interactions came together at the threefold symmetry interface (Fig. 7B). The other types of inter‐subunit interface involved the tail end of one subunit (the end with loop DE) with the side of the other at the C‐terminal half of αD, loop DE, and N terminus of αE, which covered an average of ~ 450 Å^2^. Four of such interactions met at the fourfold symmetry interface (Fig. 7C).

Although the twofold interface covered a broad surface area, it allowed some minor shift. When one subunit was aligned, the other subunit of the dimer had visible shifts, especially on the αD‐loop‐αE region, in addition to loop BC (Fig. 6B). The movement of the αD‐loop‐αE region was more pronounced in the structural superposition between Hpf‐E1 and Hpf‐E2 (Fig. 6C). This region was involved in packing against another dimer, which ensued a larger shift. However, these shifts in relative positions were relatively minor and did not impact the overall packing of the nanocages. The 24‐mer nanoparticles of Hpf‐E1 and Hpf‐E2 were essentially identical in structure.

## Discussion

In this study, we showed that it is feasible to present an array of regularly spaced *N. gonorrhoeae* antigenic peptides on the surface of Hpf by genetically inserting the peptide at the N terminus or at a loop position between Hpf helices αA and αB. The results also identified other potential insertion sites within Hpf that could be altered by mutagenesis to improve the stability and robustness of the antigen‐presenting platform. We demonstrated that inserting *N. gonorrhoeae* peptides either at the N terminus or in the middle of the Hpf sequence did not perturb the nanocage structure. Hpf is a nanocage of 24 subunits with a diameter of ~ 120 Å, which is large enough to display multiple copies of the peptides, and thus induce strong immune responses, but not so large as to present difficulties in crystallization and structural determination. Further, we showed that the chimeric proteins could be easily purified and crystallized, which will enable future rational design and improvement of the immunogens.

The idea of using Hpf as a scaffold for vaccine design was explored by Kanekiyo *et al*. 16, 17. The authors fused hemagglutinin of Influenza virus and gp350 of Epstein–Barr virus to the N terminus of Hpf. These fusion domains are larger than a single subunit of Hpf, and yet the ferritin assembled into its 24‐mer cage structure and displayed the antigenic domains on its surface. The resultant chimeras improved the potency of neutralizing antibody responses and vaccine‐induced protection against the viruses, thereby demonstrating the feasibility of using Hpf as a scaffold for rational design of vaccine immunogens.

In contrast to the hemagglutinin and gp350 domains, the *N. gonorrhoeae* surface‐exposed peptides from the outer membrane protein MtrE are relatively small. Presenting these small peptides in a large ordered array on the surface of Hpf nanoparticle is expected to increase the potency of eliciting immune responses. Equally important, using the nanoparticle as a scaffold can help present the peptides in their native conformations. Inserting the *N. gonorrhoeae* peptides in selected locations in the Hpf sequence that do not disrupt the Hpf structure, such as the Hpf‐E1 construct, can restrict the terminal positions of the inserted peptide. Keeping the peptide termini in proper close positions can promote the folding of the peptide into its native β‐hairpin structure, in principle similar to the design of synthetic loop peptides made cyclic through the addition of disulfide bonds 20, 21. To this end, we included in the inserted peptides part of the β‐strands of the outer membrane β‐barrel that flank the extracellular loops (Fig. 1D). As the entire inserted peptide is disordered in the crystal structure, it is unclear whether the peptides formed the desired β‐hairpin structure. However, judging from their sequences, which contain multiple Gly and Ser residues (Fig. 2), it is likely that the peptides can adopt many different conformations. Several studies have identified ways to stabilize β‐hairpin structure. For example, cross‐strand disulfide 22 or Trp zippers 23 can be engineered in the β‐strand linkers to promote the formation of correctly registered β‐hairpins.

It is known that β‐sheets with more strands are in general more stable 24. In this respect, the chance of presenting the *N. gonorrhoeae* antigenic peptides in their native conformation would be increased by inserting both MtrE loops in locations that are next to each other, thus allowing them to interact and form a four‐stranded β‐sheet in which their extracellular loops interact as occurs in the native MtrE structure. Alternatively, a four‐stranded antiparallel β‐sheet that displays both MtrE‐1 and MtrE‐2 loops could be designed and inserted at a single location of the Hpf sequence.

Our results also revealed that several regions of the structure have high flexibility. These flexible regions are likely related to the function of ferritin in iron oxidation and storage, such as the ferroxidase center, the fourfold symmetry channel, and the BC loop, and they can be targeted for mutagenesis to improve the stability of the nanocage structure. For the purpose of designing immunogens, it is important to maintain the stable structure of the nanocage 25, 26. The function of iron oxidation and storage does not need to be maintained. Indeed, it is desirable to produce a Hpf‐based immunogen devoid of the iron storage function to avoid affecting cellular iron homeostasis 16. The ferroxidase center has a cluster of four Glu side chains that are next to each other. Mutating some of these side chains to neutral side chains would likely increase the stability of the structure. The fourfold symmetry channel has four His side chains that coordinate a Fe ion. The loop DE that harbors the His residue has high B factors. Mutation that stabilizes this loop will likely improve the assembly and stability of the nanocage.

In conclusion, we confirmed that Hpf is a suitable platform to present *N. gonorrhoeae* peptide antigens on its surface using rational design. We identified several locations for inserting antigenic peptides in the middle of Hpf sequence, in addition to the N‐terminal fusion. The nanocage platform proved to be feasible for computational design and manageable for crystal structure determination. The results also suggested ways to improve the stability and robustness of the system and to present ordered, structurally defined antigenic loops on the nanocage surface.

## Materials and methods

### Computational design of structural models

The MtrE‐1 and MtrE‐2 structural fragments were copied from the crystal structure of MtrE (PDB code 4MT0) and placed at site 2 (His34) and site 1 (Met1), respectively, of the Hpf structure (Fig. 1), using computer 3D graphics with COOT 27. The loops were oriented to project outward from the nanoparticle surface, and carefully positioned to avoid any steric conflicts or deviations from molecular geometry requirements. The structural models were energy‐minimized and the refined models were visually inspected with COOT and checked for any geometry deviations with structural validation tools to ascertain that the main‐chain and side‐chain bond lengths, bond angles, and dihedral angles are correct.

### Protein expression and purification

The gene coding for Hpf‐E2 was synthesized (Genscript, Piscataway, NJ, USA) and cloned into pET28 (pET28‐Hpf‐E2) between *Nco*I and *Xho*I with the DNA coding for the MtrE‐2 loop peptide between *Nco*I and *Nde*I. To construct the expression plasmid for Hpf (pET30‐Hpf), a DNA fragment between *Nde*I and *Xho*I was cut from pET28‐Hpf‐E2 and inserted into pET30 between *Nde*I and *Xho*I. A sequence of 149 bp that contained the coding sequence for the MtrE loop 1 was synthesized and cloned into pET30‐Hpf between *Bsr*GI and *Hin*dIII to construct pET30‐Hpf‐E1, which encodes Hpf with the MtrE‐1 peptide replacing His34 of Hpf.

The plasmids were transformed into *E. coli* BL21 (DE3). Protein expression was induced by IPTG at 37 °C. Cells were lysed in 1 × phosphate‐buffered saline. The lysate was clarified by centrifugation. Hpf‐E2 was purified by anion‐exchange chromatography (Q HP column; GE Life Sciences, Pittsburgh, PA, USA) followed by gel filtration with a Superose 6 column (GE Life Sciences). To refold the inclusion body of Hpf‐E2, the pellet of the cell lysate was solubilized with 8 m urea in 20 mm phosphate pH 7.4, 150 mm NaCl, and the protein was refolded by removing urea through stepwise dialysis in the same phosphate buffer with additional 4, 2, and 0 m urea. The refolded Hpf‐E2 protein was then purified by gel filtration. Both refolded and the soluble Hpf‐E2 proteins eluted from Superose 6 column at the same position. Hpf was purified by a one‐step ammonium sulfate precipitation followed by gel filtration. Hpf‐E1 was purified by a one‐step ammonium sulfate precipitation followed by gel filtration and hydrophobic interaction chromatography (butyl HP column; GE Life Sciences).

### Electron microscopy

The purified protein samples were negatively stained with phosphotungstic acid, and the images were taken on a JEOL JEM‐1011 transmission electron microscope. Electron micrographs were taken for Hpf, Hpf‐E1, and Hpf‐E2. All three proteins gave identical particles of ~ 12 nm on the electron micrographs under the experimental conditions.

### Protein crystallization, data collection, and structural determination

Protein crystallization was carried out using the sitting drop vapor diffusion method. The crystals of Hpf‐E1 were obtained from a precipitant solution containing 20% glycerol, 1.6 m NaCl, and 8% PEG 6000. The Hpf‐E2 crystals were obtained from 1.7 m (NH_4_)_2_SO_4_ and 0.1 m Tris pH 8.0. Crystals were directly mounted from the drops and frozen in liquid nitrogen.

X‐ray diffraction data were collected at −140 °C on a Rigaku 007 X‐ray diffraction system with a Raxis 4++ detector. Data were indexed, integrated, and scaled with HKL2000 28. Data processing statistics are listed in Table 1. The scaled data sets were converted to amplitudes, and a randomly selected subset (5%) of data was set aside for cross‐validation during structural refinement, using the programs in the CCP4 Suite 29.

The structures were determined by molecular replacement with Phaser 30, using the crystal structure of Hpf (3BVF) to build search models. For Hpf‐E1, a six‐subunit model was used, whereas a four‐subunit model was constructed for Hpf‐E2. Structural refinement was performed with Refmac 31 with automatic local NCS restraints and isotropic B factor refinement. The last few rounds of refinement were carried out with the TLS refinement. The Hpf‐E2 crystal was twinned, and twin refinement was carried out after the R factors were lower than 35%. Structural visualization, manual building, and corrections were carried out using Coot. Structural refinement statistics are listed in Table 1.

## Data accessibility

The atomic coordinates and structure factors are available in the Protein Data Bank under the accession codes 5U1A and 5U1B. 5U1A is the structure of Hpf‐E1 with the MtrE loop 1 replacing His34 of Hpf; 5U1B is the structure of Hpf‐E2 with the MtrE loop 2 fused to the N terminus.

## Author contributions

SW and AEJ designed the experiments. LW and SW prepared the constructs and purified the proteins. DX and SW set up crystallization experiments. SW collected the X‐ray diffraction data and determined the crystal structures. ALV was involved in the manuscript preparation. SW and AEJ wrote the manuscript.
